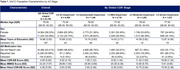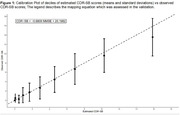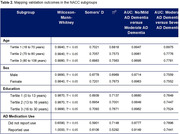# Mapping between the Mini‐Mental State Examination and the Clinical Dementia Rating scale for Patients with Alzheimer’s Disease: An External Validation Study

**DOI:** 10.1002/alz.087285

**Published:** 2025-01-09

**Authors:** Niels Juul Brogaard, Julie Hviid Hahn‐Pedersen, Jens Gundgaard, Pepa Polavieja, Benjamin D Bray, Mei Sum Chan, Men Hoang, Daniel Araya, Dominic Trepel

**Affiliations:** ^1^ Novo Nordisk A/S, Søborg Denmark; ^2^ Lane Clark & Peacock LLP, London United Kingdom; ^3^ Health Analytics, Lane Clark & Peacock LLP, London United Kingdom; ^4^ Trinity College Dublin, Dublin Ireland

## Abstract

**Background:**

The Clinical Dementia Rating scale Sum of Boxes (CDR‐SB) measure is commonly used in specialist research settings and clinical trials. In clinical practice, shorter cognitive assessments, such as the Mini‐Mental State Examination (MMSE), are commonly used. A targeted literature search identified existing MMSE – CDR‐SB mappings; such as Balsis *et al*. 2015 and Perneczky *et al*. 2006. However, neither mapping provided granular translation of these measurements across all Alzheimer’s disease (AD) stages. This study therefore aimed to extend and externally validate existing published mappings between the MMSE and CDR to support the translation of measurements between these tools across all AD stages.

**Method:**

The optimal functional form for the mapping was investigated using power‐transformed univariate regressions, and the modelled mapping was extended to capture the full range of AD stages. External validation of the mapping was conducted using the US National Alzheimer’s Coordinating Centers (NACC) database, stratified by age, sex, education, and AD medication use. Individuals’ most recent visit with valid MMSE and CDR‐SB scores, and concordant AD staging based on clinical diagnoses and global CDR scores were included. Calibration and discrimination metrics were reported, following best practice guidelines for clinical prediction tool validation.

**Result:**

A linear MMSE to CDR‐SB mapping (see Figure 1 for equation) was found to have the optimal functional form and demonstrated high goodness of fit (Adjusted = 0.989). When externally validated on the NACC population (n = 25,768; Table 1), the mapping satisfied all validation metrics (Somers’ D = 0.705, R^2^ = 0.739; above the prespecified 0.7 threshold), and deciles of patients by CDR‐SB were generally well‐calibrated (Figure 1). The mapping also demonstrated acceptable discrimination between no/mild AD dementia versus moderate AD dementia (AUC = 0.885; 95%CI: 0.879‐0.891) and between moderate AD dementia versus severe AD dementia patients (AUC = 0.756; 95%CI: 0.743‐0.769). The mapping accurately predicted AD stage across the overall NACC database and when stratified by subgroups (Table 2).

**Conclusion:**

This extended mapping for MMSE to CDR‐SB demonstrated good performance, especially in less severe AD stages. The findings can support the translation of AD severity assessments between datasets using CDR‐SB and MMSE, bridging a common information gap between clinical trials and real‐world evidence studies.